# Tests and analyses on physical and mechanical properties of fresh black fungus in picking season

**DOI:** 10.1371/journal.pone.0275565

**Published:** 2022-10-13

**Authors:** Shuaiyang Wang, Weidong Song, Mingyou Wang, Jiaoling Wang, Tianhang Ding, Dehuan Zhou, Shixin Ma

**Affiliations:** 1 National Edible Fungus Industry Technology System Mechanization Laboratory, Nanjing Institute of Agricultural Mechanization, Ministry of Agriculture and Rural Affairs, Nanjing, Jiangsu, China; 2 School of Mechanical and Electrical Engineering, Nanjing University of Aeronautics and Astronautics, Nanjing, China; University of Vigo, SPAIN

## Abstract

This study determined the physical and mechanical characteristics of fresh black fungus during the harvesting season to provide basic data for the development of mechanical equipment for black fungus harvesting and processing. We have conducted a comprehensive test of black fungus cultivars “Heishan”. The mono-factor separation force experiments of black fungus and black fungus virgulate medium were conducted. It was noted that the tension angle was an important factor affecting the separation force, which was mainly distributed between 1.06 and 3.65 N. Besides, the average value of Poisson’s ratio of black fungus was measured to be 0.445 in the tensile test of black fungus leaves using image recognition and analysis techniques, with a test error within 2.5%; and the average value of tensile elastic modulus and shear elastic modulus of black fungus leaves was 0.947 MPa and 0.327 MPa, respectively; we also found that the average tensile strength at the root of black fungus was not significantly different from that at the leaf, which was around 0.436 MPa. In addition, it was obtained that the height and thickness dimensions of black fungus in the picking season conformed to a normal distribution, and concentrated around 34.39mm and 0.92mm respectively.

## Introduction

Black fungus, known as wood ear, jelly ear, Auricularia auricula, belongs to edible Auriculariales fungus [1, 2], which can grow between 6°C and 36°C. Globally, black fungus is mostly concentrated in China, Japan, Korea, and Vietnam in the Asia-Pacific region [3]. China is the first country in the world to cultivate black fungus due to its location in the northern temperate zone, with a mild climate and abundant rainfall. Its history of artificial cultivation has been more than 1300 years. Also, China is currently the main production country of black fungus in the world and is the main exporter of black fungus worldwide. In 2019, China’s production of black fungus reached 7.018 million tons, an increase of 4.12% year-on-year, accounting for more than 90% of the world’s total production [4]. And the annual production steadily increased to 7.0643 million tons in 2020 [5]. With the development of black fungus cultivation technology, Chinese black fungus production technology has roughly gone through four stages: natural wild cultivation, log chopping for cultivation, log inoculation cultivation, and poly bag cultivation [6].

Poly bags method is an efficient method for artificial cultivation of black fungus in China. According to local natural conditions, Chinese people use corn stalk, mulberry sawdust, cottonseed shell, etc. instead of natural wood resources as the raw materials of black fungus culture medium, which effectively improves the yield of black fungus and effectively alleviates the ecological pressure. In the cultivation mode of poly bag, the black fungus virgulate medium was shortened to the black fungus stick in this paper because of the cylindrical shape. The sticks of black fungus are hung in the greenhouse or grown on the field. The former is not yet widely used due to its high cost. And the latter is the main method of cultivation at present. In the field planting method of black fungus, there are dilemmas such as high artificial harvesting costs and low level of mechanized harvesting [7]. And when processing fresh black fungus, the processing machinery does not work well due to the lack of the necessary physical and mechanical parameters of black fungus. In order to solve the technical problems of mechanized harvesting and processing of black fungus, it is necessary to study the physical and mechanical properties of black fungus in the harvesting season. This can provide reasonable suggestions for combining edible fungus machinery with edible fungus agronomy, also provide a theoretical basis for the research and development of black fungus harvesting and processing equipment, and indicate the direction for machine simulation optimization.

At present, scholars all over the world have carried out systematic theoretical research on the characteristics of crops and vegetables, and have determined the parameters of size, shape, structure and mechanical properties as well as the interrelationships of each parameter. Jakob, M.C. and M. Geyer selected the robot picking method of cucumbers by measuring the fruit removal forces of cucumbers [8]. Guocheng Bao tested the mechanical properties of sweet potato and obtained the collision loss mechanism of sweet potato, which provided a theoretical basis for the development of sweet potato harvester [9]. ILkur Alibas and Nezihe Koksal measured the physical, mechanical and structural characteristics of seeds of three Turkish pepper varieties [10]. Wenshuo Gao conducted a tensile test study on Flammulina velinata, which provided a basis for the formation design of picking machinery and the selection of picking part [11]. Shen Haiyang studied the mechanical and physical characteristics of sweet potato blocks in harvest period, which provided basic theoretical support for the design of key components and the determination of operation parameters of sweet potato combined harvester [12]. Li Hongbo studied the tensile mechanics of foxtail millet stem leaf sheath leaf and its binding position, and obtained the variation rule of mechanical parameters along the stem internodes [13]. Yingjie Guo measured the shear strength and tensile strength of agaric virgulate medium, providing a design basis for the development of fungus sticks crushing machinery [14]. Kovács, Á. and G. Kerényi studied the physical and mechanical properties of corn stalk in combination with the working principle of corn harvesting machinery [15]. Akshay Sonawane tested the physical, thermal and mechanical properties of Bael fruit, providing a basis for the development of fruit processing machinery [16]. Shengtao Jia studied the shape and extrusion characteristics of Garlic from Xinjiang, which provided a theoretical basis for garlic grading and precision seeder optimization [17]. Thulasimani Krishnakumar determined the physical and mechanical properties of short and long-duration cassava cultivars to a peeling machine [18]. These researches provide a data basis for the simulation experiments of harvesting and processing machinery and equipment, which made the mechanized equipment of crops more targeted, greatly accelerates the research and development speed of the machine, and improves the work quality and efficiency.

However, so far, no scholars have studied the physical and mechanical properties of black fungus. And the development of mechanized harvesting and processing equipment for black fungus is facing a big technical bottleneck. To address this problem, we learned testing methods from the previous mechanical testing of cucumber, agaric virgulate medium, pepper seeds, Flammulina velutipes, sweet potatoes, millet stems and leaves, garlic, corn stalks, Bael fruit, cassava cultivars, etc. And we took the black fungus of "Heishan" as the research object, determined the density, height, thickness, elastic modulus, Poisson’s ratio, size, and weight of the fungus sticks, etc. The basic formula of material mechanics and elastic mechanics was used to calculate the shear modulus of black fungus leaves, and a detailed comparative analysis was carried out, which provided theoretical support for the harvesting and processing equipment of fresh black fungus.

## Materials and methods

Black fungus sticks were provided by the Tonglu County Nongfa Grain and Oil Professional Cooperative in Hangzhou, Zhejiang Province, China, and the inoculated variety of the fungus stick is "Heishan". In 2021, during the black fungus harvesting season, 10 sticks were randomly selected from the field using a double diagonal five-point sampling method [19] and marked with a red marker.

**Fig 1:**
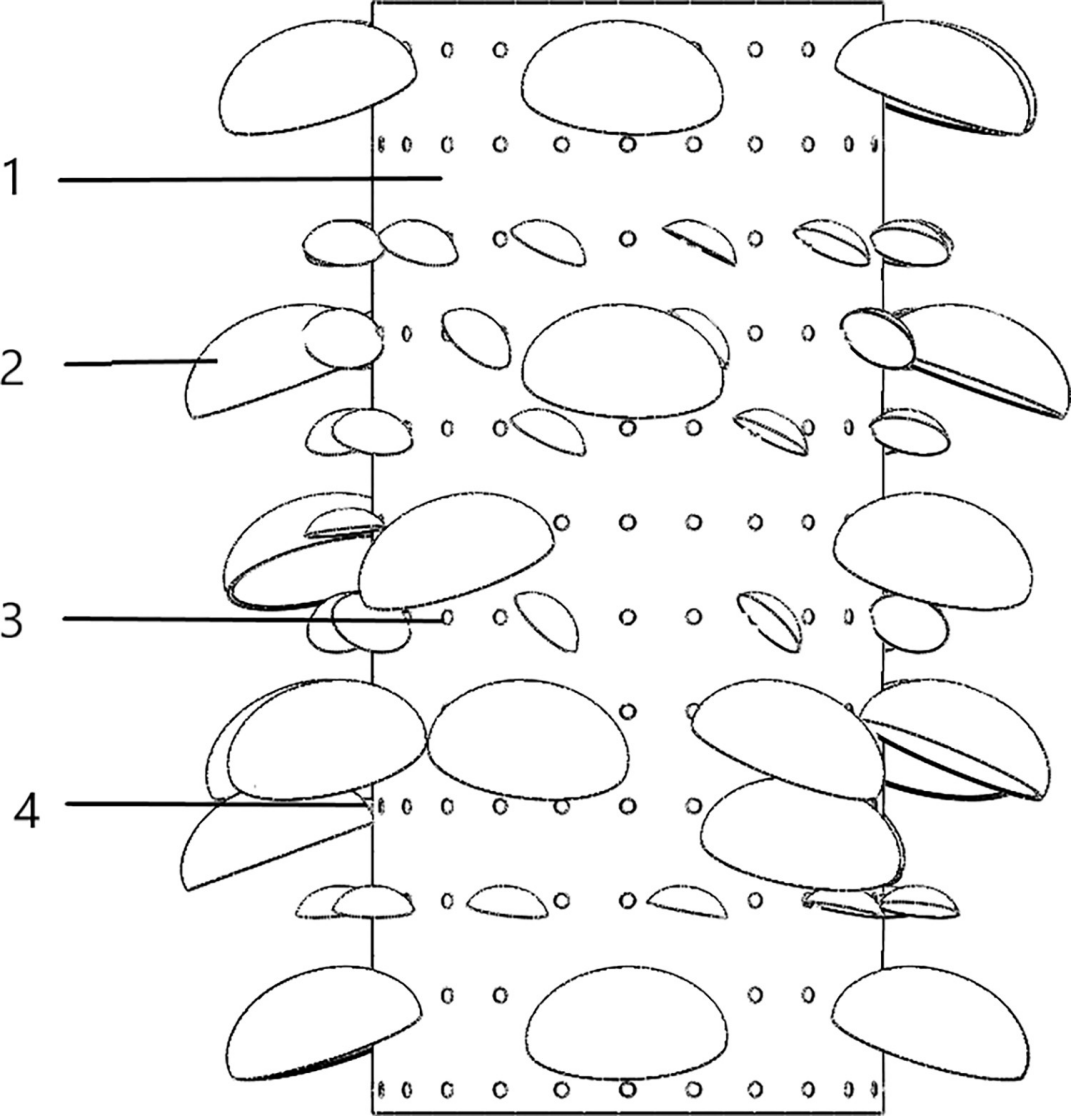
Model of black fungus leaves, puncture holes and black fungus sticks. 1. Black fungus sticks 2. Black fungus leaves 3. Puncture hole 4. the base of the black fungus leaves.

**Fig 2:**
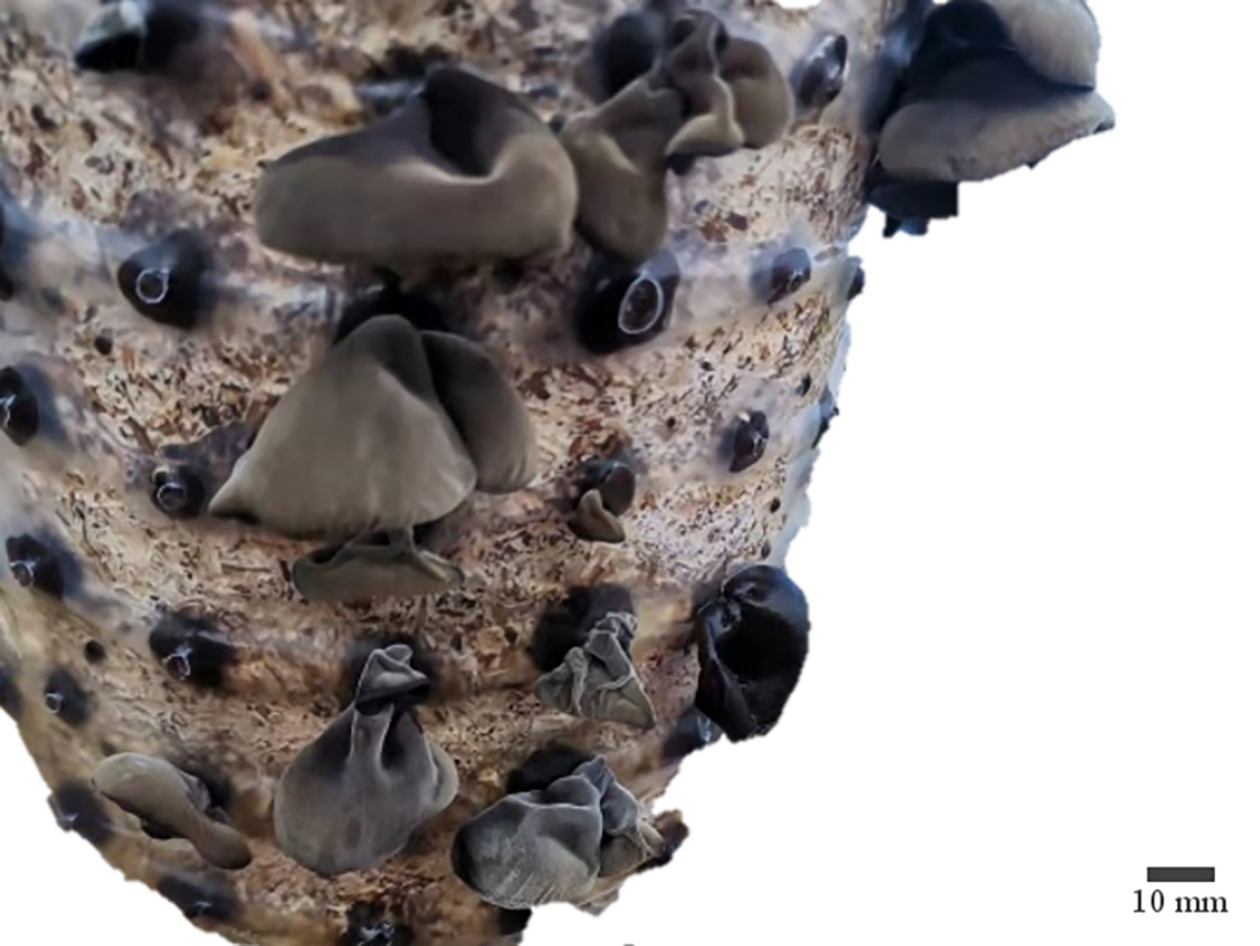
Physical picture of black fungus.

**Fig 3:**
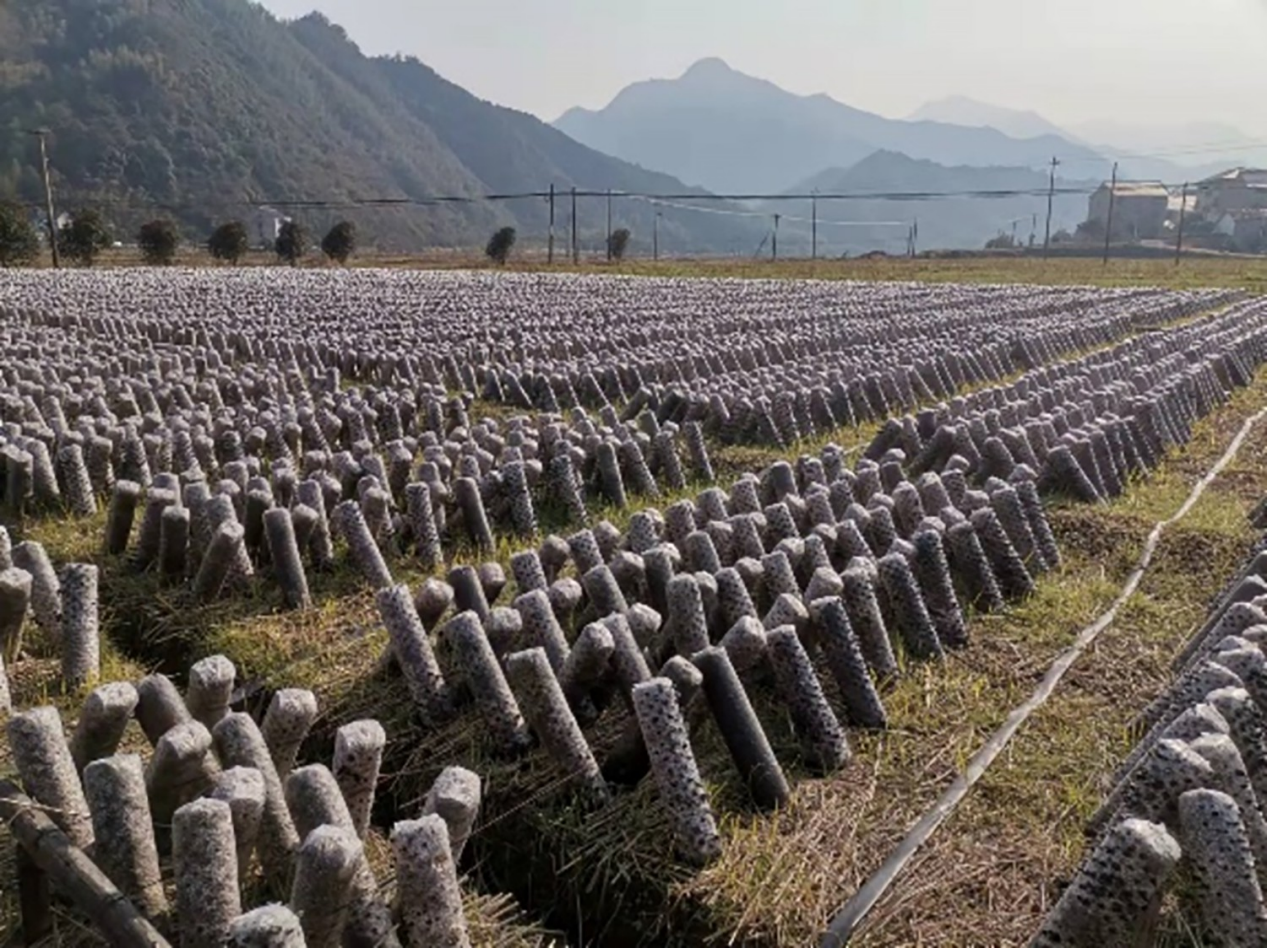
Black fungus sticks field.

The black fungus bags were of standardized size 15x55cm, with a height of 41cm and a diameter of 9.5cm after filling with culture material. And it has an average weight of about 1.78kg. Its puncture method is circular, and the puncture hole diameter is about 3mm. The black fungus field, black fungus sticks, puncture holes, and black fungus leaves are shown in Figs 1–3. The experiment was completed in the Mechanics Laboratory of Nanjing Institute of Agricultural Mechanization, Ministry of Agriculture and Rural Affairs. All statistical analyses were performed using the Origin2018 software package.

In the mechanics of materials, the material parameters such as stress, strain, the tensile modulus of elasticity, and Poisson’s ratio can be calculated from the force-displacement curve obtained by the tensile test [20]. It mainly involves the following six formulas:

$$\sigma =\frac{F}{A},$$ (1)

where: *σ* –tensile stress (N/mm^2^), F–tensile force (N), A–The cross-sectional area in the vertical tensile direction (mm^2^).

$$\varepsilon =\frac{\Delta l}{l},$$ (2)

$$\varepsilon^{\prime }=\frac{\Delta b}{b},$$ (3)

where: Δ*l* –displacement in tension direction (mm), *l* –length (mm), *ε* –longitudinal strain, Δ*b* –lateral displacement (mm), b–width (mm), *ε*–lateral strain.

$$E=\frac{\sigma }{\varepsilon },$$ (4)

$$\upsilon =-\frac{\varepsilon^{\prime }}{\varepsilon },$$ (5)

$$G=\frac{E}{2\;(1+\upsilon )},$$ (6)

Where: E–elastic modulus (MPa), *υ* –Poisson ratio, *G* –shear modulus (MPa).

The moisture content of black fungus leaves was measured using the DHS-20A electronic moisture tester produced by Bonsey Instrument Technology (Shanghai) Co. According to the relevant drying technical regulations of black fungus [21], the drying temperature was set to 70°C and the drying time was 150 min. The measurement principle is to determine the moisture content by calculating the mass ratio before and after drying:

$$\eta =\frac{M_{1}-M_{2}}{M_{1}},$$ (7)

Where: *η* –moisture content (%), *M*_1_–Weight before drying (g), *M*_2_–Weight after drying (g).

The black fungus mono-factor separation force test, Poisson’s ratio test, and black fungus leaf tensile test are all taken in the universal material testing machine. The machine was the DWD type electronic universal material machine produced by Shenzhen Suns Company. Its rated load is 5KN and the error is within 0.5%. The displacement resolution is 0.01mm and the loading rate is 0.01~300mm/min. The force, displacement, and other parameters during the test can be automatically recorded by the software that comes with it.

**Fig 4:**
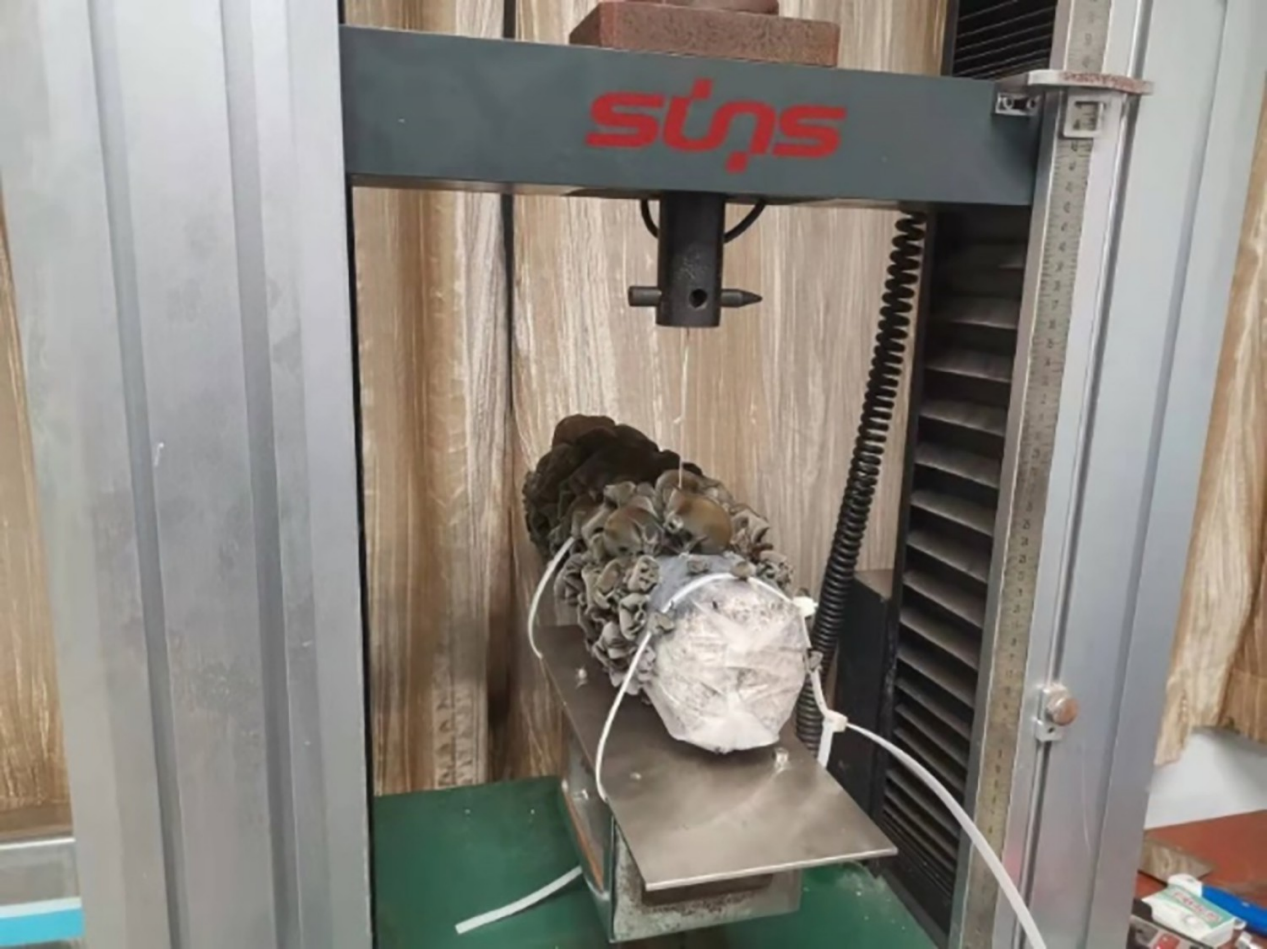
The test of separation force.

In the mono-factor separation force test of black fungus, the black fungus sticks to be picked were selected and marked with a marker. And the sticks were fixed on the test machine fixture using the self-locking nylon tie. The black fungus leaves were clamped using a pair of strong magnets, which were connected to the universal material testing machine through a non-elastic rope, as shown in Fig 4. To simplify the test analysis, the four influencing factors of black fungus height, loading rate, tension angle, and moisture content were labeled as A, B, C, and D. Five replicate tests were conducted at each level, and the test results are shown in Tables 1–5. Immediately after the separation force measurement, the moisture content was measured.

**Table 1:** Variance Analysis of separation force at different loading speeds.

Source of variance	Quadratic sum	Degree of freedom	Mean square	F value
Factor A	0.07545	2	0.03773	0.5393
Error	0.83944	12	0.06995	--
Total	0.91489	14	--	--

**Table 2:** Variance Analysis of separation force between the sticks and black fungus of different heights.

Source of variance	Quadratic sum	Degree of freedom	Mean square	F value
Factor B	12.0956	2	6.0478	141.5906
Error	0.5126	12	0.0427	--
Total	12.6082	14	--	--

**Table 3:** Variance Analysis of separation force at different tension angles.

Source of variance	Quadratic sum	Degree of freedom	Mean square	F value
Factor C	1.7911	2	0.8955	6.6837
Error	1.6078	12	0.1340	--
Total	3.3989	14	--	--

**Table 4:** Separation force of black fungus and the sticks with different moisture content.

D	Separation force (N)	The mean of separation force (N)	The mean of strength (MPa)	Moisture content of black fungal root and leaf (%)
D_1_	3.29、3.07、2.86、3.28、3.13	3.126	0.442	85.7、86.2
D_2_	2.69、3.45、3.04、3.28、2.95	3.083	0.436	84.8、82.0
D_3_	3.33、3.17、3.21、3.46、2.92	3.218	0.455	84.1、78.3
D_4_	2.89、3.45、3.29、2.57、3.64	3.168	0.448	82.5、72.5
Mean	--	3.149	0.445	--

**Table 5:** Variance Analysis of separation force between black fungus and the sticks with different moisture content.

Source of variance	Quadratic sum	Degree of freedom	Mean square	F value
Factor D	0.0507	3	0.0169	0.1950
Error	1.3864	16	0.0867	--
Total	1.4371	19	--	--

In the experiment on the effect of black fungus height on separation force, black fungus height was the vertical distance from the top of the black fungus leaf to the fungus stick. According to the growth of black fungus and the relevant harvesting standards [22], we divided the black fungus height (A) into three intervals: 0–25 mm immature black fungus (A_1_), 25 mm to 40 mm mature black fungus (A_2_), and over-mature black fungus above 40 mm (A_3_). The loading rate (B) was selected as 15mm/min (B_1_), 45mm/min (B_2_), and 75mm/min (B_3_) for the test according to the conditions of the universal material testing machine. The tension angle (C) was the angle between the line of force action and the growth direction of black fungus in the cross-section of black fungus sticks at 0° (C_1_), 45° (C_2_) and 90° (C_3_), respectively. In the test of the effect of moisture content (D) on the separation force, sampling groups were taken at 2 h (D_1_), 8 h (D_2_), 18 h (D_3_), and 28 h (D_4_) after water spraying, so as to control the water content of black fungus. In the mono-factor separation force test, factors other than the test independent variables were set as follows: the separation force test was conducted at 8 hours after water spraying, the mature black fungus with a height between 25 and 40 mm was selected, the pulling loading rate was set to 15mm/min, and the tension angle was°.

**Fig 5:**
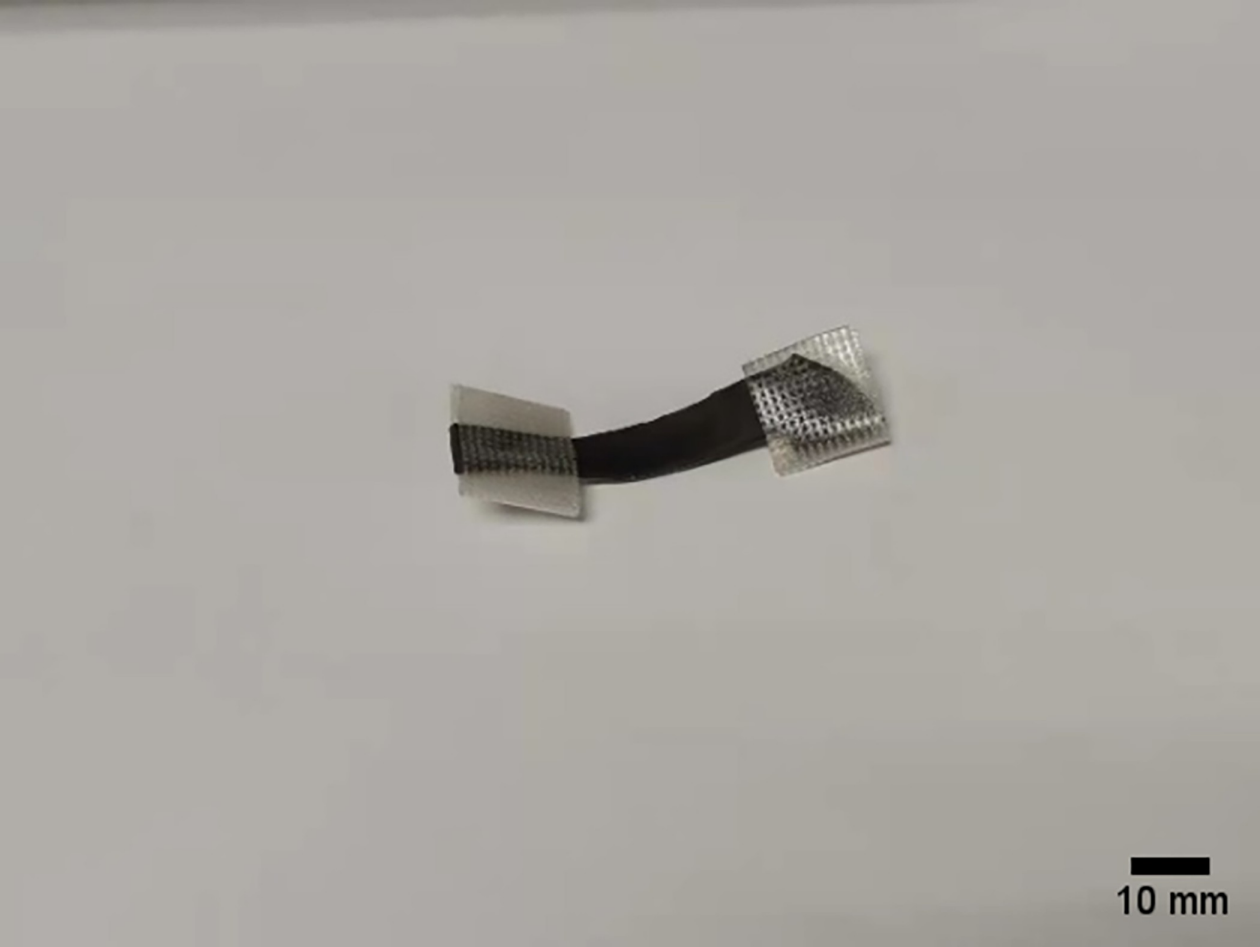
Specimen of a black fungus leaf.

**Fig 6:**
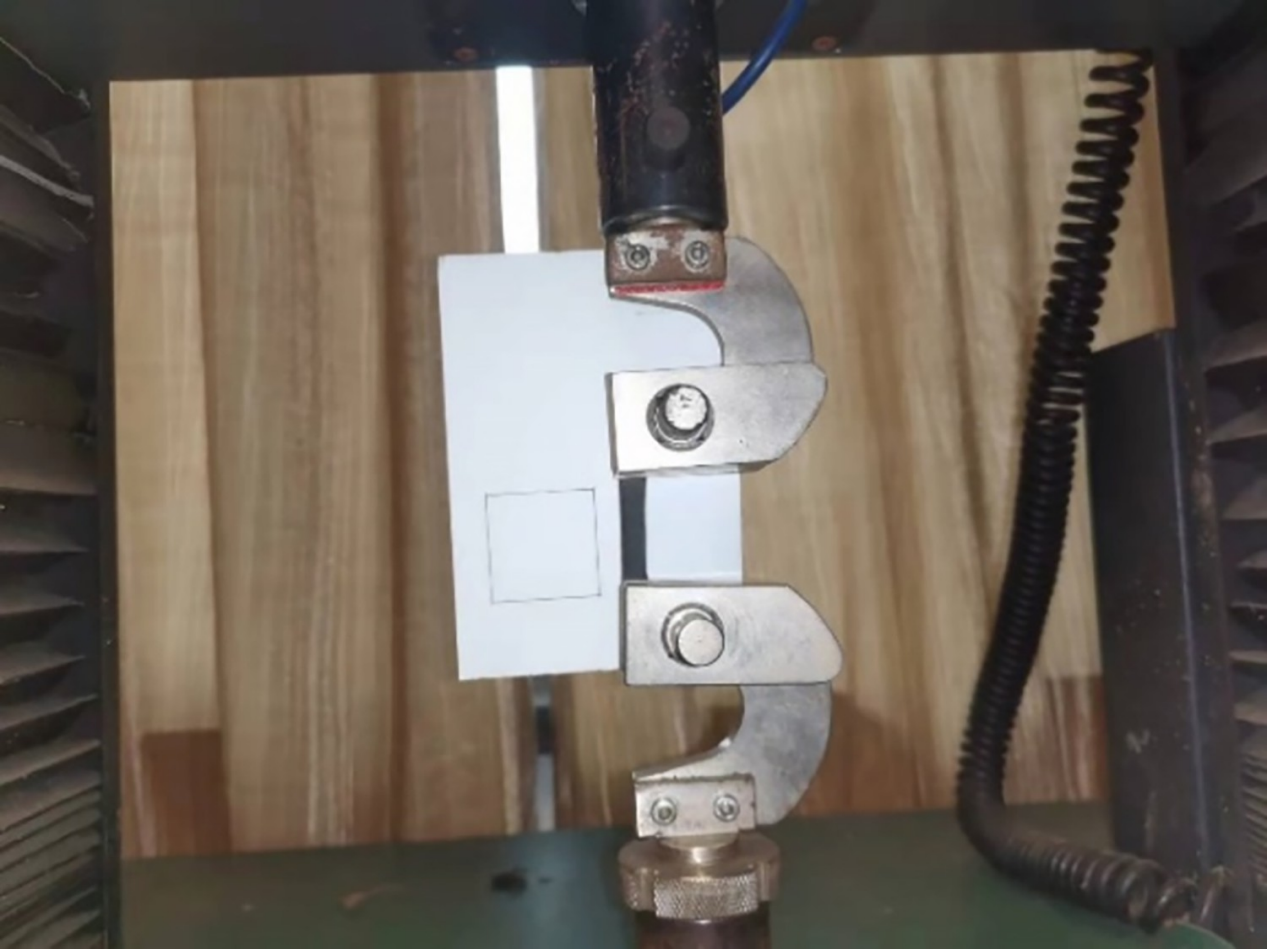
Tensile test of a black fungus leaf.

Freshly harvested mature black fungus leaves were selected for tensile and Poisson’s ratio measurement tests on black fungus leaves. The black fungus leaves were made into specimens with an effective length of 15–30 mm and a width of 4–8 mm using a utility knife. Both ends of the specimen were wrapped with PE medical adhesive tape to increase the friction force between the specimen and the fixture. The specimens are shown in Figs 5 and 6. Tensile, density and Poisson’s ratio measurement tests were performed on the black fungus leaves. These tests were repeated five times, and the moisture content was measured immediately once after the tests.

The loading speed of the universal material testing machine was set to 15 mm/min during the tensile test of the black fungus leaves. Video recording was performed using a high-speed camera of the FASTEC IMAGING Hispec5 model manufactured by Pisan (Shanghai) Photoelectric Technology Co. A Falconeyes CLL-1600TW spotlight was used for lighting the environment. The high-speed camera was adjusted to the same height as the specimen and faced the specimen plane during the test to ensure that the test error is within a reasonable range.

**Fig 7:**
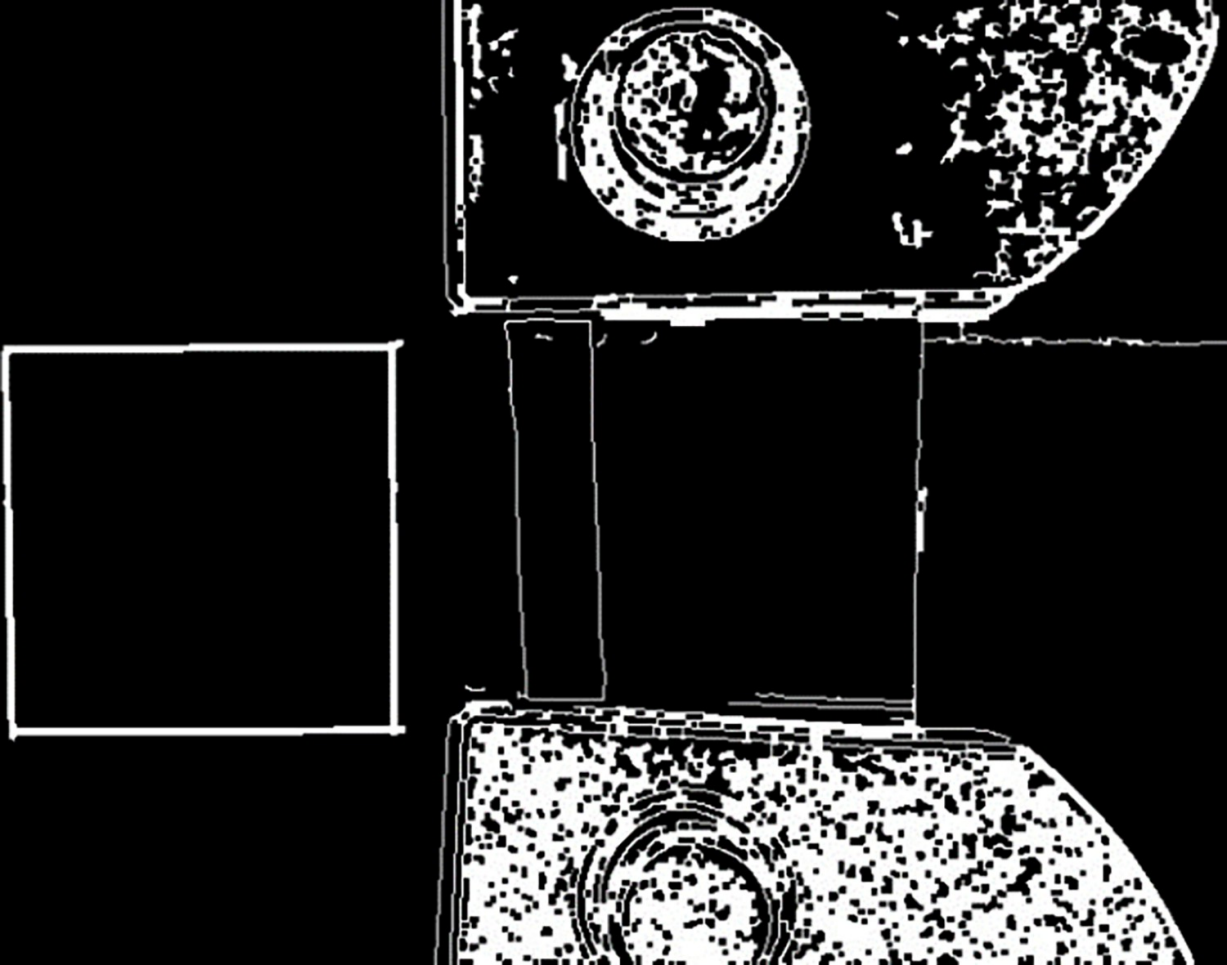
Extraction of edges.

**Fig 8:**
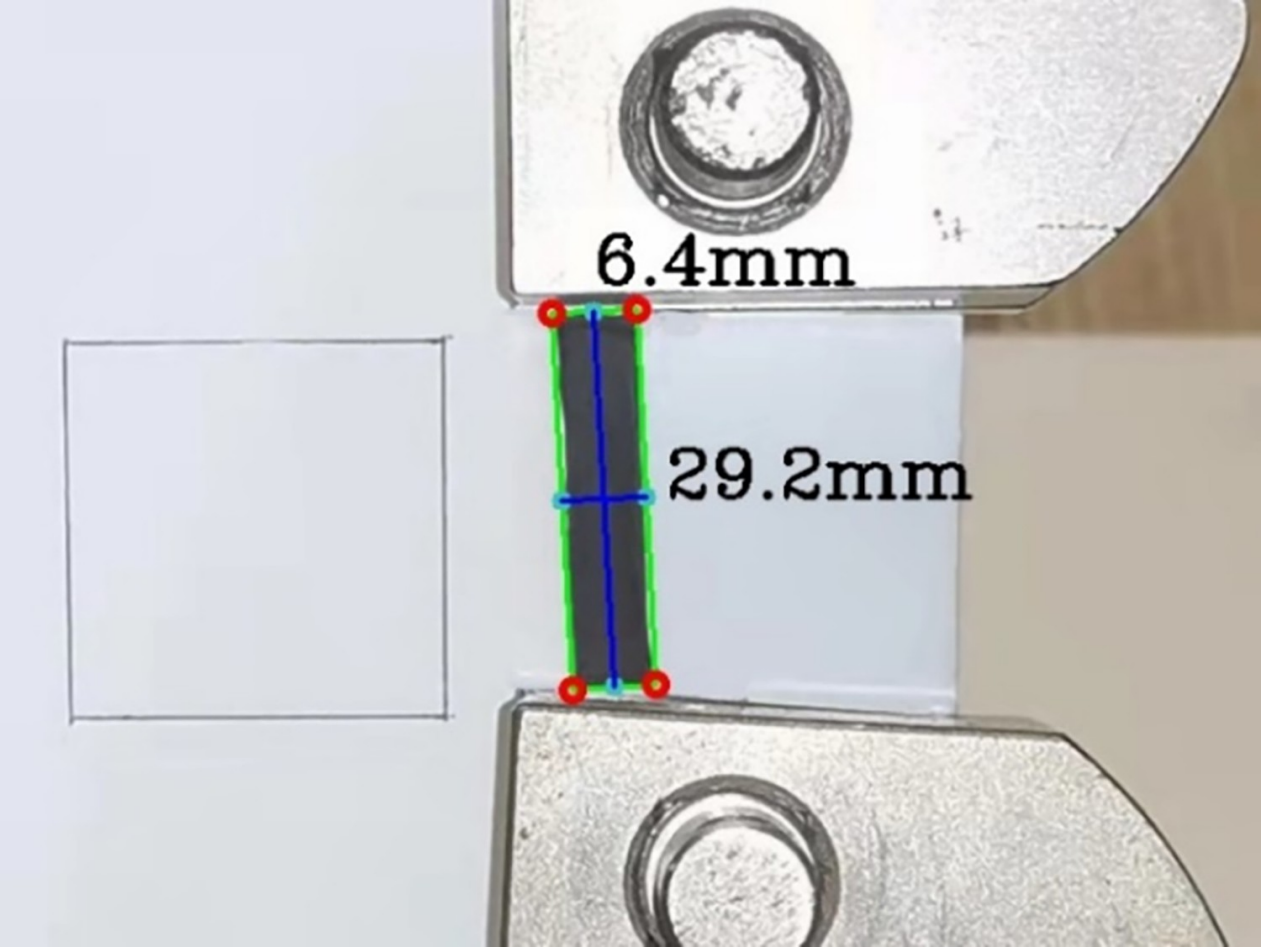
Size calculation.

The measurement and calculation of Poisson’s ratio of black fungus was accomplished using high-speed camera technology in combination with the image recognition and analysis software platform Python-Opencv [23, 24]. In the black fungus leaf tensile test, the image-recognition analysis platform Python-Opencv software platform was used to write the code, and the main operations such as conversion to grayscale map, Gaussian filtering, extraction of edges, contour processing, and size calculation were performed on the images, as shown in Figs 7 and 8. The principle of scale measurement was as follows: take the 30mm square on the left as the reference benchmark, the measurement of other contours within the picture was carried out; and the measurement of transverse and longitudinal displacement of black fungus was completed by combining the scale changes before and after the black fungus tension, so as to derive the transverse and longitudinal strain; and the Poisson’s ratio of the black fungus leaf was obtained by using the Poisson’s ratio calculation formula.

**Fig 9:**
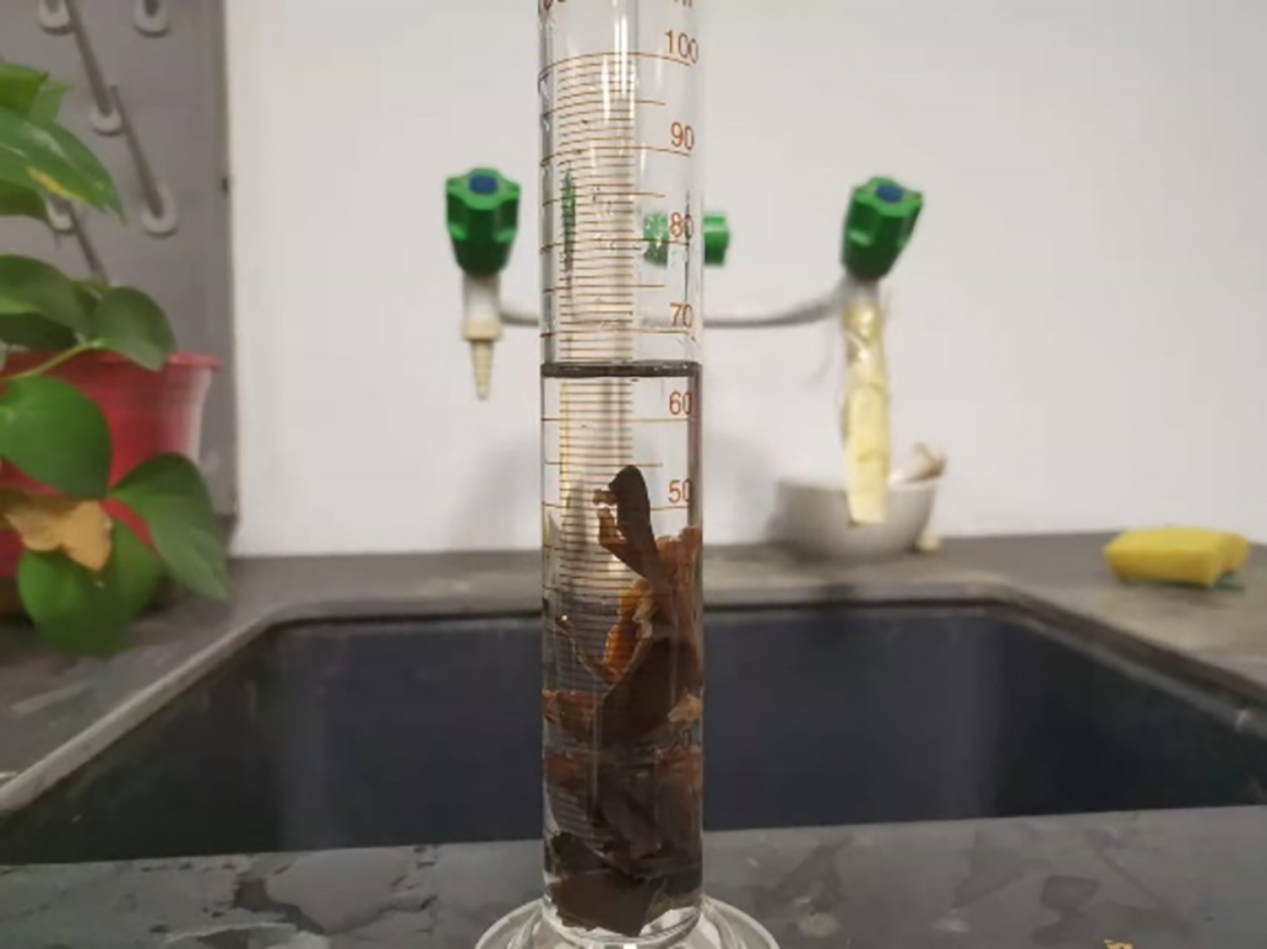
Density test of black fungus.

Since black fungus is denser than water, the density test was measured using the drainage method [25], as shown in Fig 9. Mature black fungus that had been sprayed with water for 8 hours was selected and the water content was measured to be 84.1%. The black fungus was cut with scissors into small pieces that could be completely immersed in a graduated cylinder. To prevent water absorption, the black fungus was coated with petroleum jelly and then the density was measured by weighing each of the five samples using an electronic balance. After being placed into a 100 ml graduated cylinder, once the liquid level was stabilized, the readings were taken immediately. The accuracy of electronic balance and graduated cylinder in the test was 0.1g and 0.1ml respectively, both of which had high accuracy.

## Results and discussion

**Fig 10:**
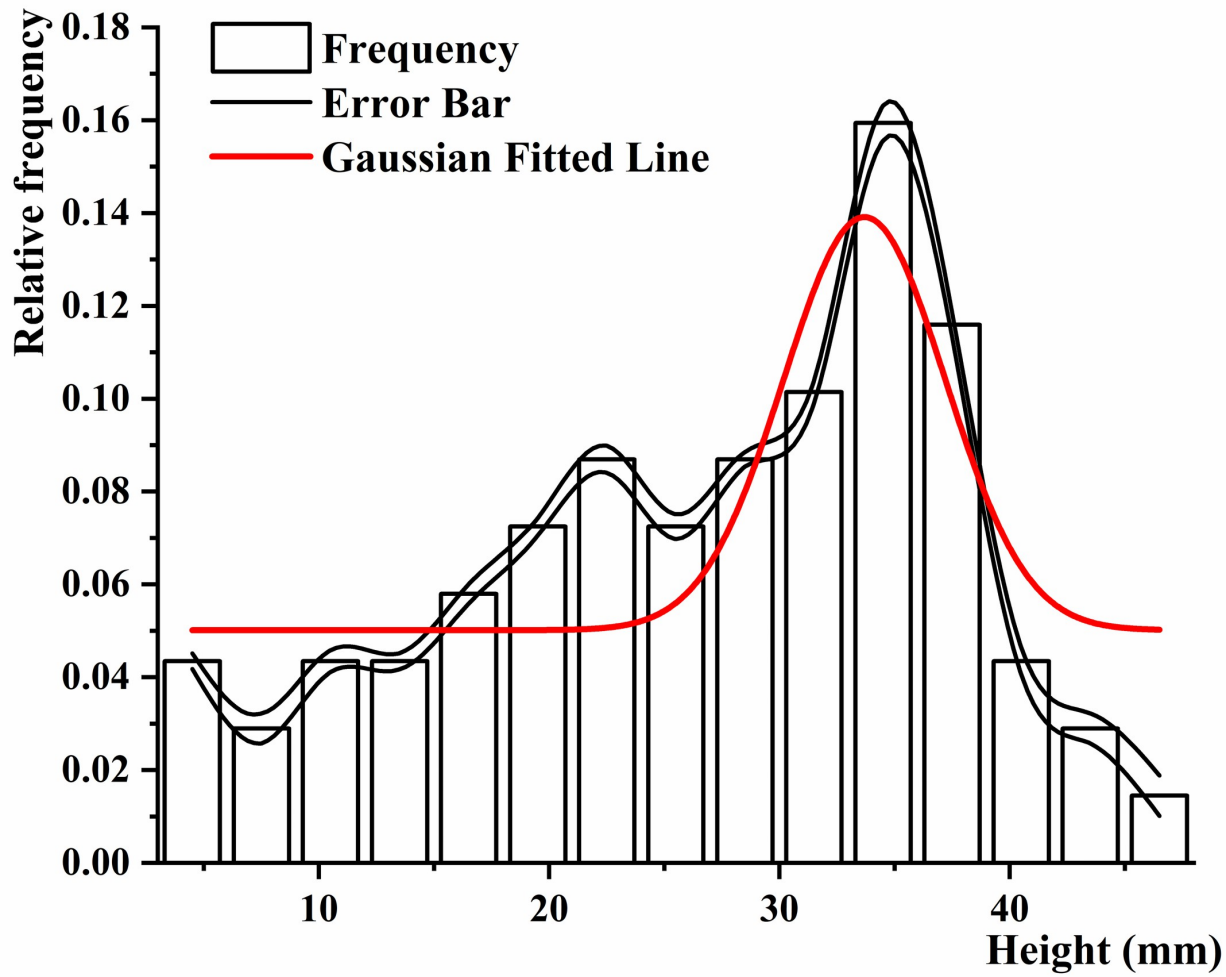
Height distribution of black fungus.

**Fig 11:**
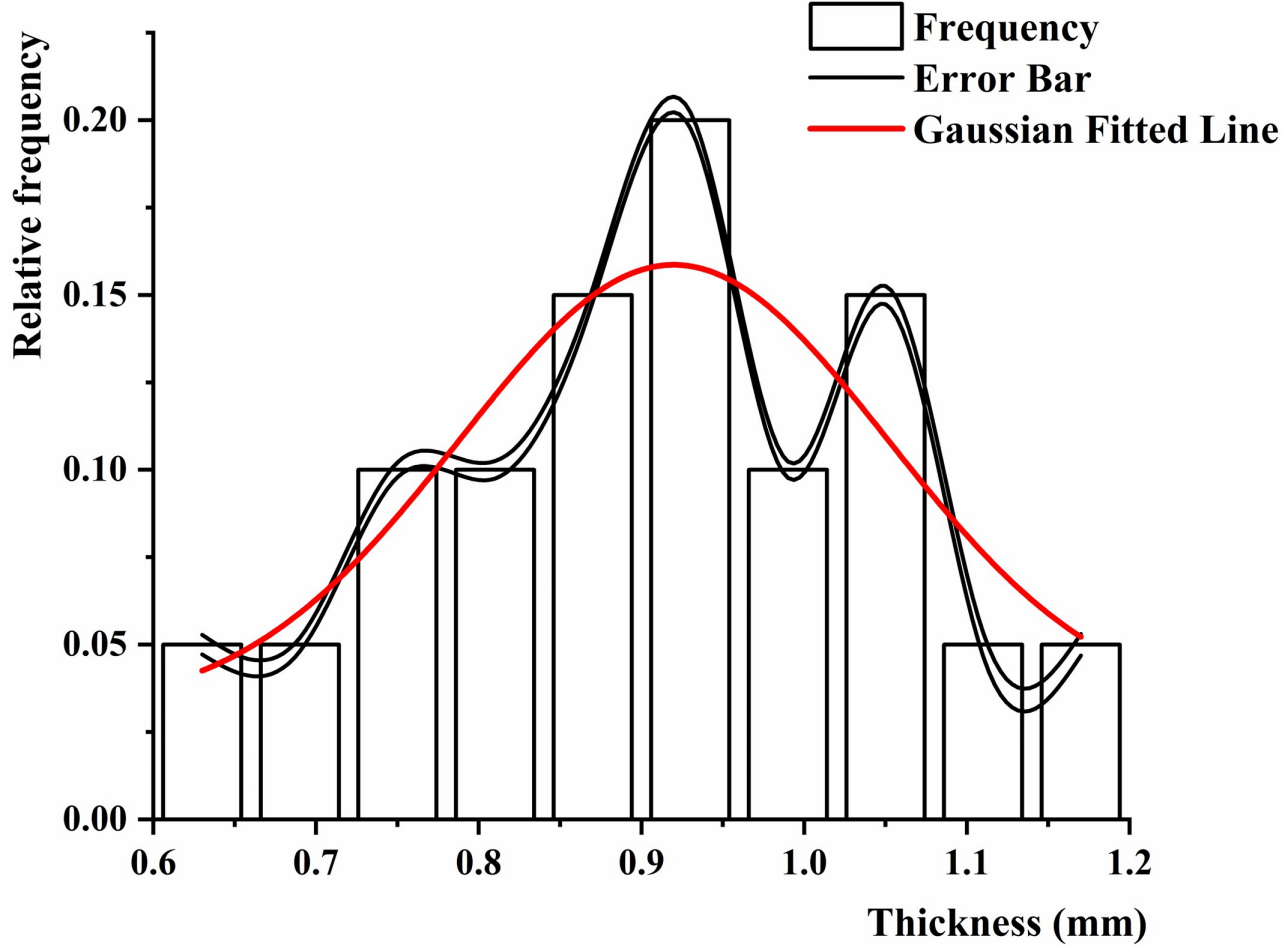
Thickness distribution of black fungus.

Data on height and thickness of the black fungus can be used to develop automated harvesting equipment and processing machinery for the black fungus harvesting industry. We selected two black fungus sticks at random to measure the height of 138 black fungi after 8 hours of water spraying; 20 mature fungus leaves were selected and measured with a digital vernier caliper. A frequency histogram was drawn to perform curve fitting. It can be seen from Figs 10 and 11 that the height and thickness of mature black fungus conform to the normal distribution, the expected μ is 34.39 mm, 0.92 mm, and the standard deviation σ is 5.87 and 0.22, respectively.

**Fig 12:**
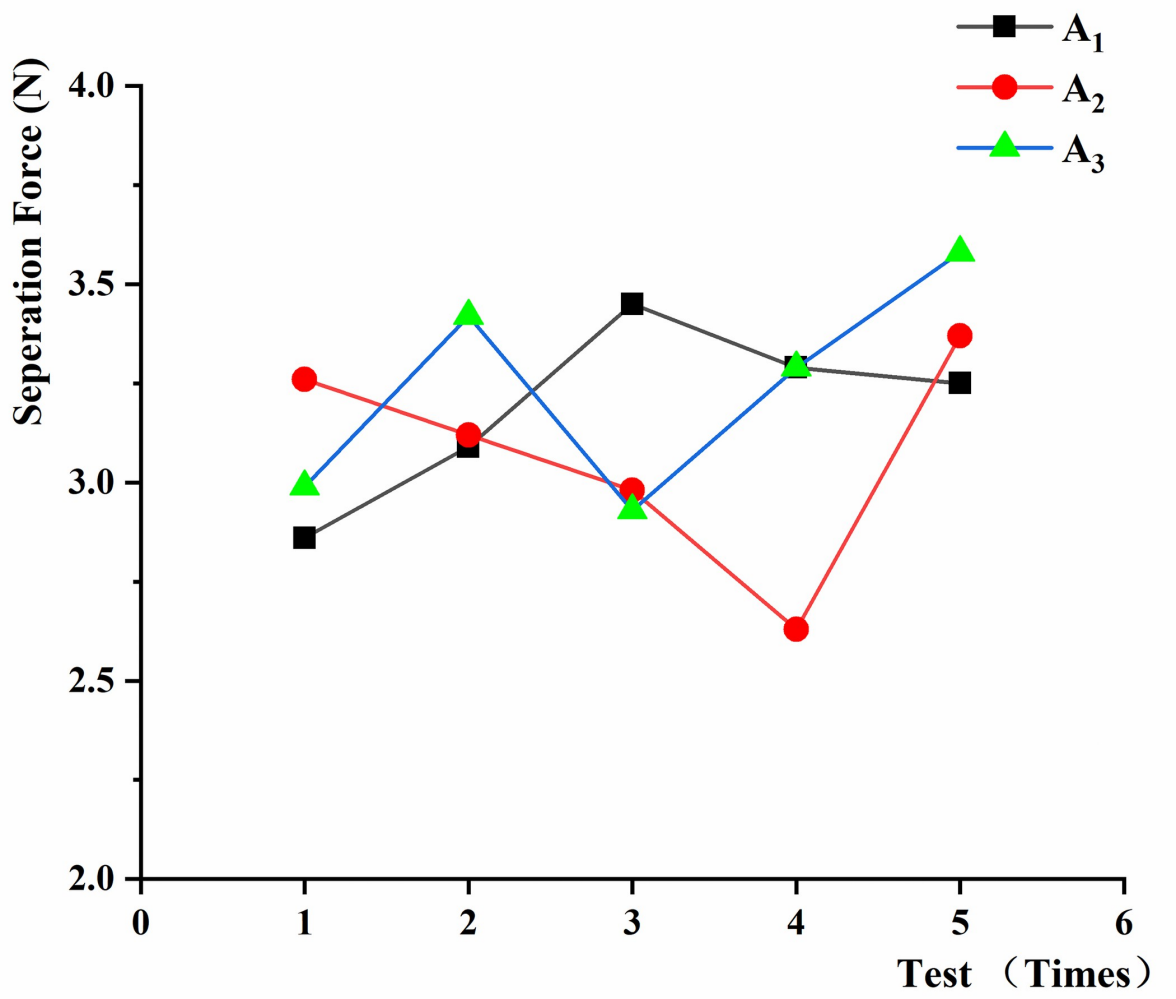
Separation force between black fungus and the sticks at different loading speeds.

**Fig 13:**
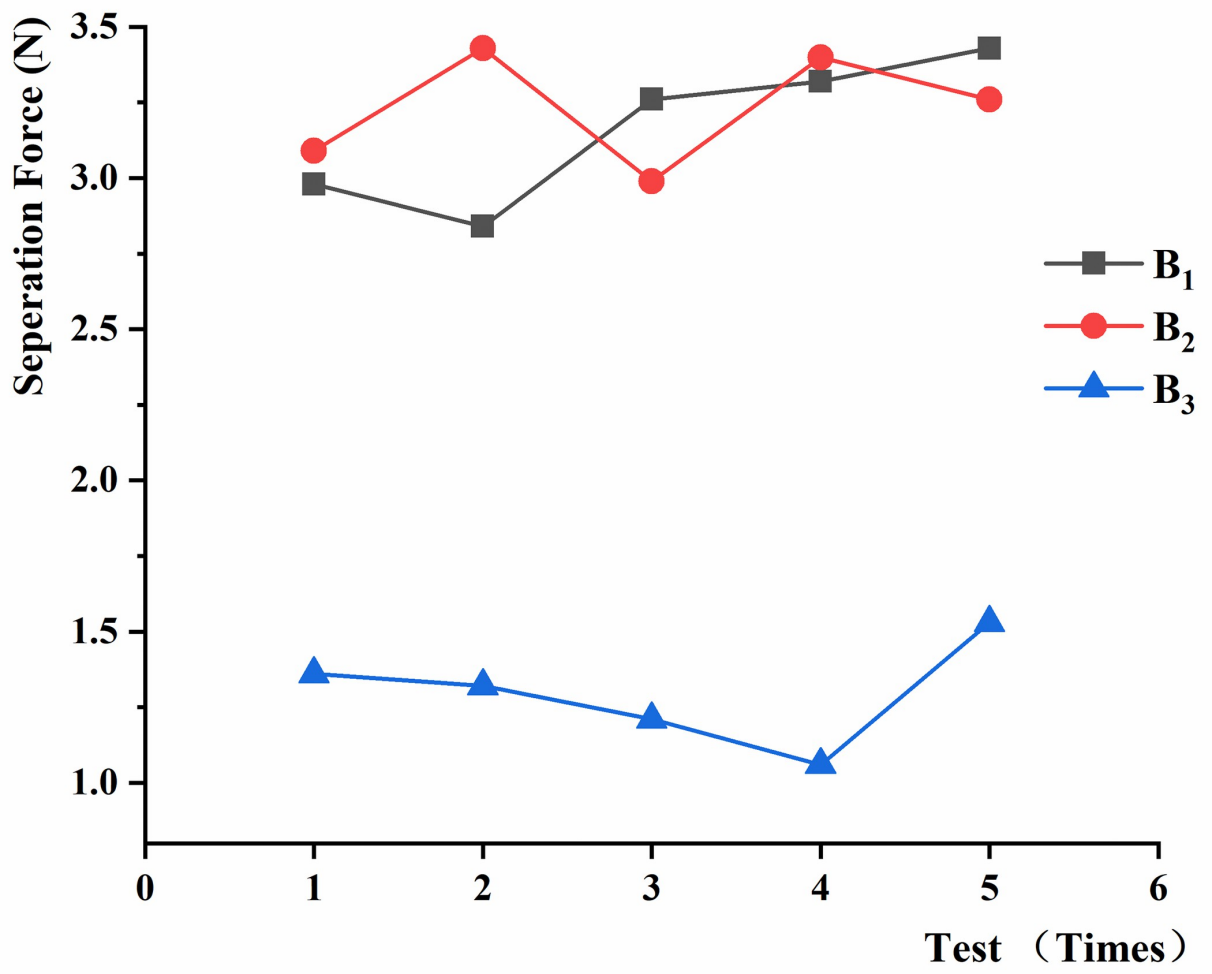
Separation force between the sticks and black fungus of different heights.

**Fig 14:**
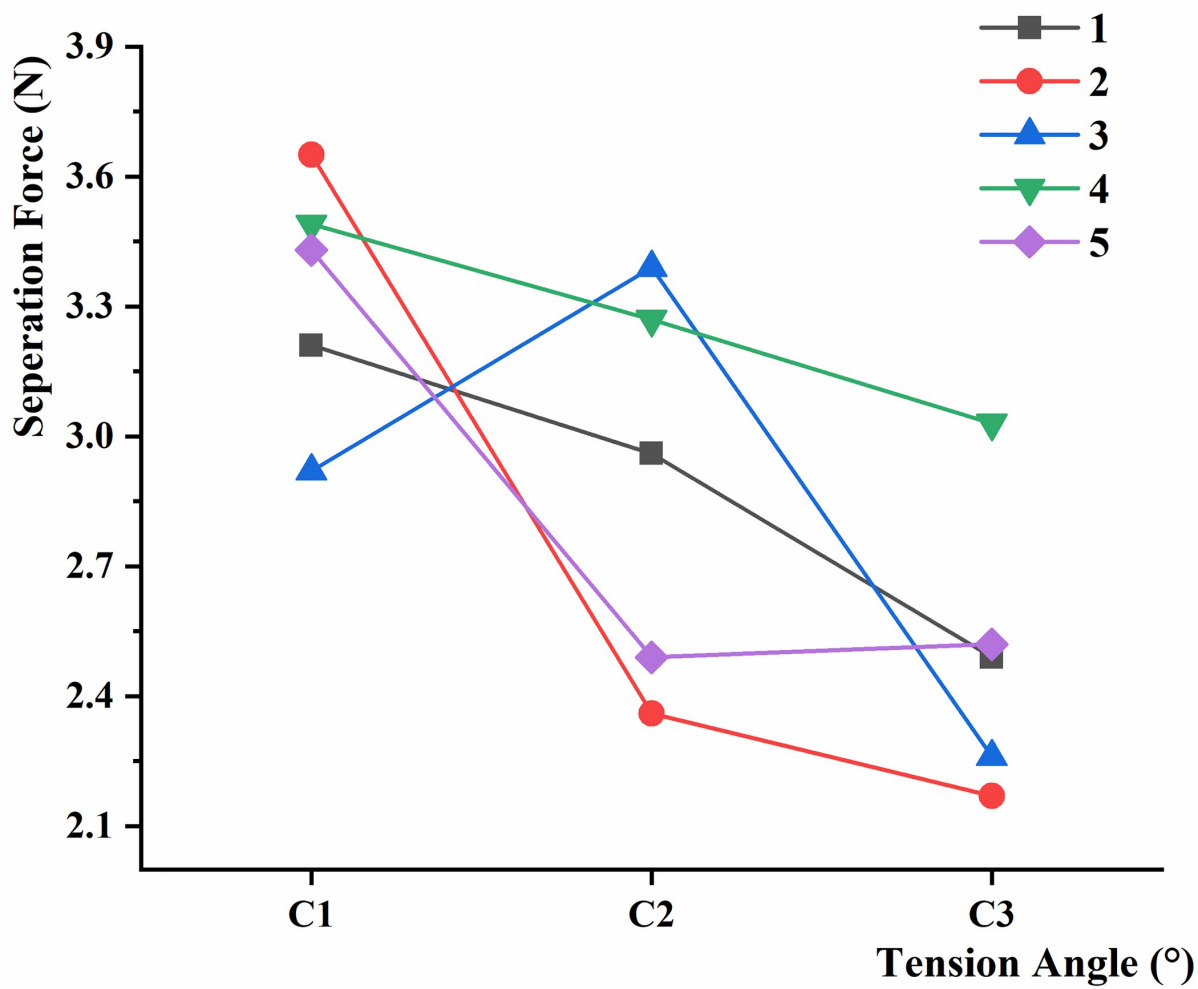
Separation force between black fungus and the sticks at different tension angles.

In the test of the separation force between black fungus and the sticks, the black fungus tensile fracture failure could be mechanically modeled as the cylindrical shape, due to the cylindrical shape of the puncture holes. After removing the invalid data, the results and analysis are shown in Figs 12–14. It was found that the required displacement for separation varied between 7.6 mm and 13.7 mm because of the different positions of magnet clamping.

**Fig 15:**
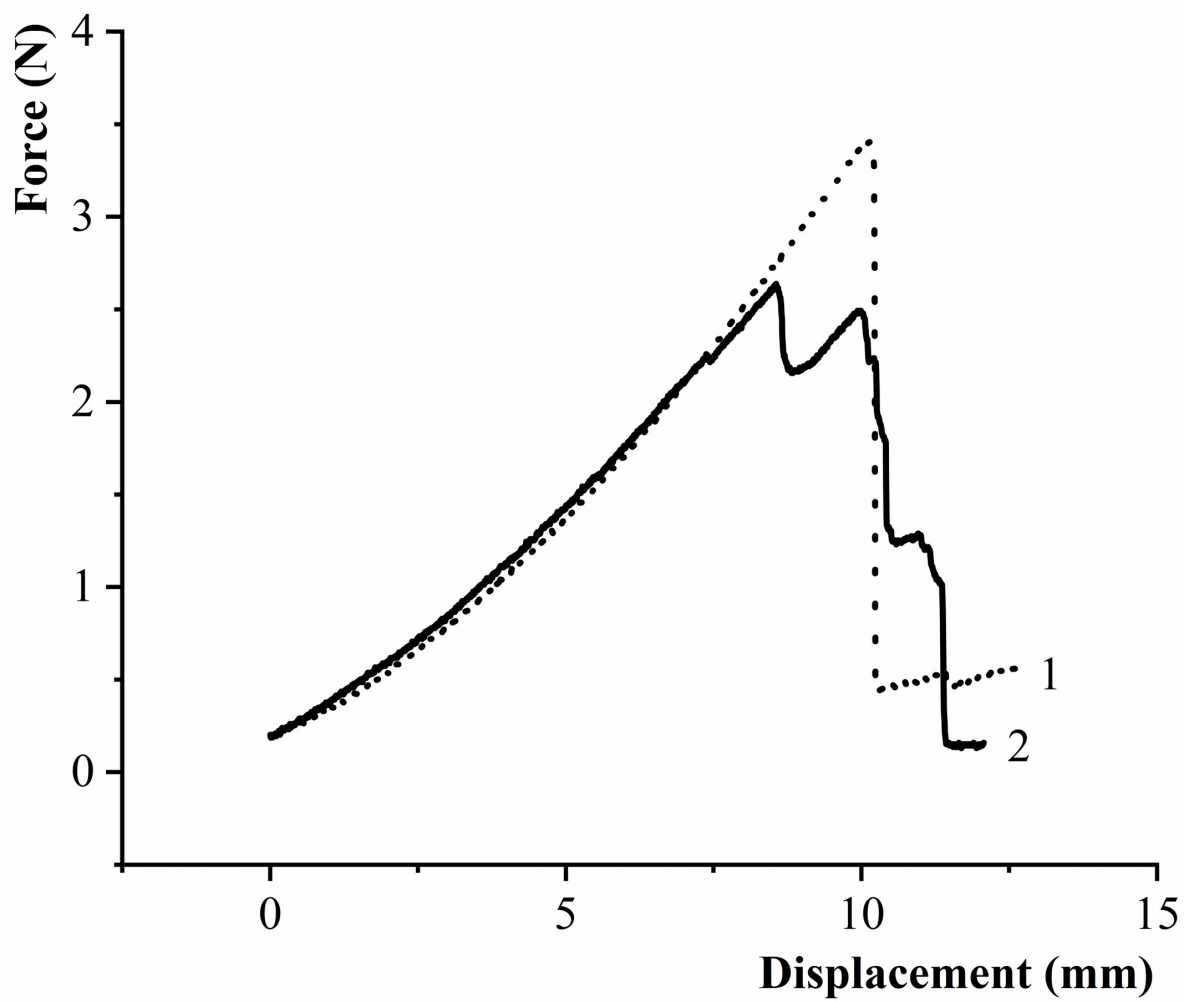
Typical force-displacement curve of black fungus separation force test.

During the pulling process, due to the high connection force between black fungus and substrates, the fracture failures all occurred near the puncture hole of the bag, the ear base area. There are two typical fracture modes, as shown in Fig 15. The specific performance is as follows: the first is that the early stage in the process of fracture failure is elastic deformation, and after reaching the failure threshold, the crack occurs suddenly, as shown in curve 1 in Fig 15; the second failure mode is that: after reaching a certain force value, due to stress concentration, multiple tears occur at the base of the ear, and the failure form is as shown in Fig 15, curve 2. Under the two fracture modes, the tensile force and displacement showed a linear change trend at the early stage of drawing, which was in line with the mechanical properties of elastic materials, and there was no obvious yield phenomenon at the base of black fungus leaves.

In the analysis of equal repeated ANOVA for the data of black fungus separation force tests, the joint hypotheses test method [26] was used for analysis, as shown in Table 1. In the test of loading rate for separation force, the significant level α was set to 0.05. And F = 0.5393, we are over 95% certain that the loading speed does not have a significant effect on the separation force between black fungus and the sticks. We can consider that the separation force was measured in a quasi-static state between 15 and 75mm/min of loading rate.

**Fig 16:**
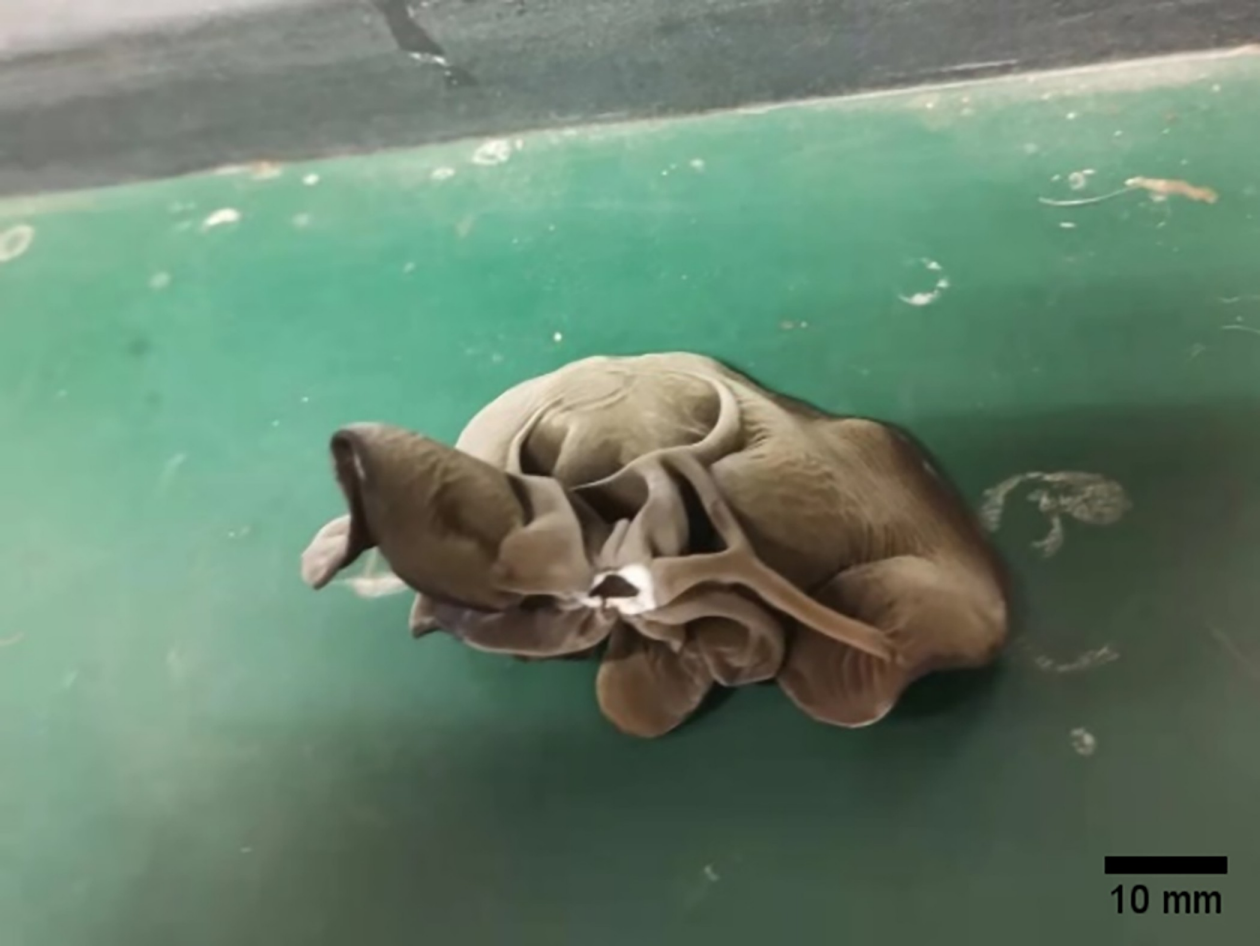
The over-mature black fungus.

In the separation force test on black fungus height, the significant level α was set to 0.05. F = 141.5906, as Table 2 shown. According to this, we are over 95% certain that the height has a significant effect on the separation force between black fungus and the sticks; by LSR multiple comparison analysis [27], the conclusion was that there is no significant difference in the separation force between the immature black fungus and the mature black fungus, while the separation force of the over-mature black fungus is different, significantly decreased compared to the other when the significant level α = 0.05. Observing the fracture at the base of the black fungus leaves, as shown in Fig 16, the periphery of the leaves base has been partially corrupted, and the leaves base has become significantly thinner, resulting in a decrease in the separation force between the black fungus leaf and the stick.

In the separation force test of the tension angle, F is equal to 6.6837, at the significant level α = 0.05. The Table 3 shows more details. We can conclude that the average separation force is related to the angle of pulling force acting on the growth direction of the black fungus. And the tension angle is deemed to have a significant effect on the separation force, just like the removal forces of peanut [28]. This can provide direction for the optimal design of the black fungus harvesting machine.

In the moisture content test of the separation force between black fungus and the sticks, the moisture content of the leaf was measured to be about 83%, after 8 hours of water spraying. In this case, the average tensile resistance of separation was 3.149N, the standard deviation σ between groups was 0.05, the mean tensile strength was 0.436MPa, as shown in Table 4. The tensile failure of the separation force test between the black fungus and the sticks mainly occurred at the base of the leaf. During the water spray management of black fungus in the picking season, the moisture content at the base of the leaf and the rest of leaf was quite different. And the moisture content at the base of the leaf dropped less than the rest. The tensile resistance and tensile strength of different groups of black fungus are nearly the same.

In the separation force test of the moisture content, F is equal to 0.1950, at the significant level α = 0.05. The Table 5 shows more details. Therefore, it can be considered that the water spray management of black fungus during the picking season has little effect on the separation force between black fungus and fungus sticks. However, for the drying process and other processing technique of black fungus, it is recommended that the leaf be harvested after 8 hours of water spraying.

The density of the mature black fungus picked 8 hours after water spraying was measured. After removing the invalid data, it was determined that the density of the mature black fungus of the "Heishan" variety was between 1.087 and 1.096 g·cm^-3^, with an average value of 1.091 g·cm^-3^. The results are shown in Table 6.

**Table 6:** Density test data of black fungus.

Specimen	M/g	V_1_/cm^3^	V_2_/cm^3^	*ρ*/(g·cm^-3^)
1	6.541	60	66.0	1.090
2	5.675	60	65.2	1.091
3	4.892	60	64.5	1.093
4	7.023	60	66.5	1.087
5	4.384	60	64.0	1.096
Mean	5.704	60	65.24	1.091

**Fig 17:**
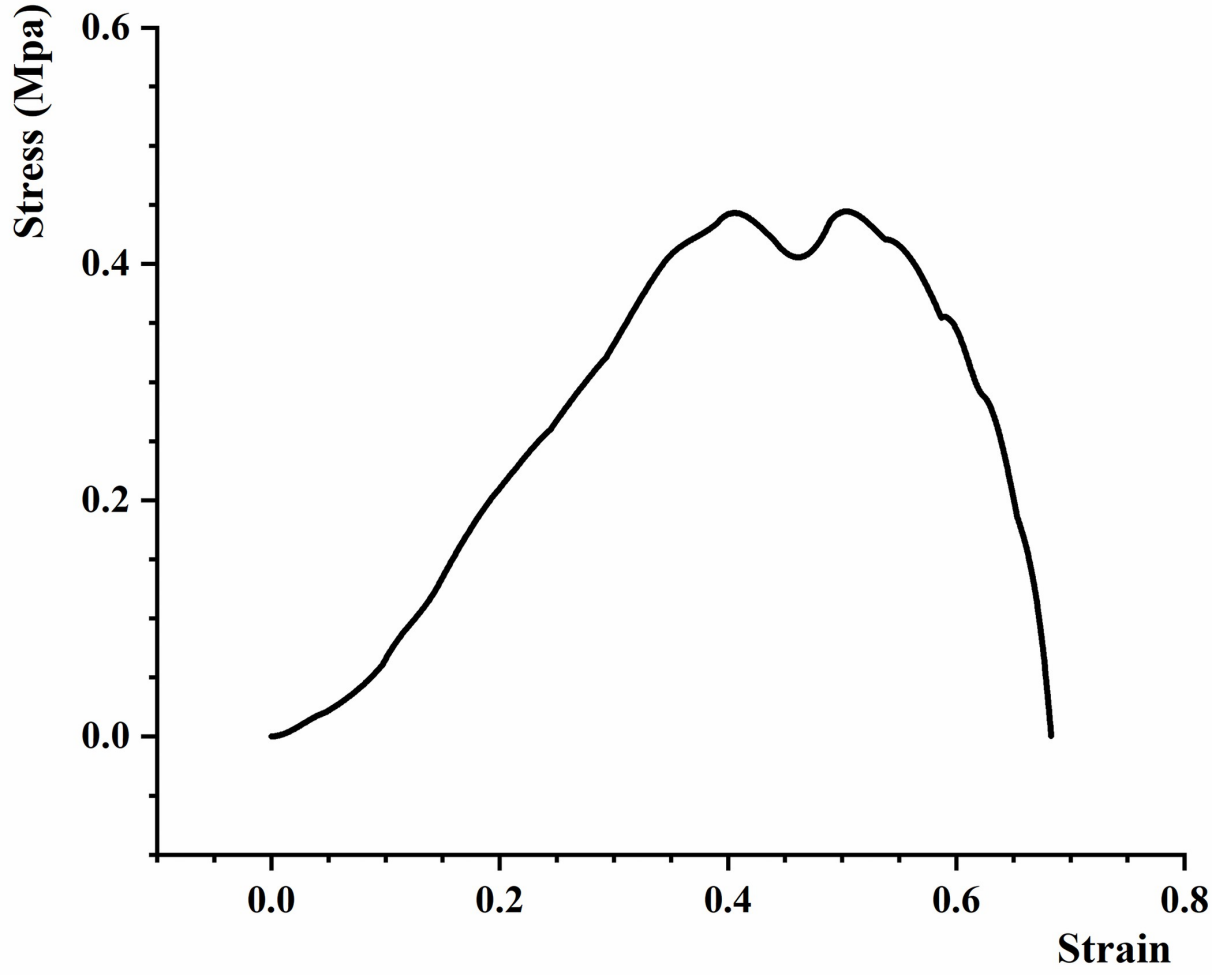
A typical stress-strain curve.

Combined with the tensile test records of black fungus leaves and the image recognition displacement measurement results, the average values of tensile strength, elastic modulus, Poisson’s ratio, and shear modulus were 0.454 MPa, 0.947 MPa, 0.463 and 0.327 MPa, respectively. The results are shown in Tables 7 and 8. And a typical stress/strain curve of black fungus leaves is shown in Fig 17. In Poisson’s ratio measurement test, we made a check test between the displacement recognition results in the length direction and the truth value results from the distance sensor of the universal material testing machine, because the transverse displacement truth value is difficult to measure. The control trial showed that the measurement error was less than 2.2%, which meets the requirements for the Poisson’s ratio of fruits and vegetables [29]. The standard deviations of strength, elastic modulus, Poisson’s ratio, and shear modulus are 0.028, 0.070, 0.019 and 0.055, respectively. And the coefficient of variation are 0.062, 0.074, 0.043 and 0.167. They indicate that the degree of dispersion between sample data is mall and the data are reliable. These physical and mechanical properties can help the processing machine of black fungus.

**Table 7:** Recorded data of Poisson’s ratio measurement test of black fungus leaves.

Spec-imen	Length, width, thickness (mm)	Longitudinal displacement from image recognition (mm)	Real longitudinal displacement (mm)	Reco-gnition error (%)	Transverse displace-ment (mm)
1	20.6、7.9、0.91	8.6	8.73	1.5	1.5
2	25.8、4.2、0.84	13.3	13.59	2.2	0.9
3	25.2、5.2、0.69	13.3	13.54	1.8	1.2
4	29.3、6.1、0.95	11.1	11.27	1.5	1.1
5	22.1、3.9、0.76	12.8	13.02	1.7	1.0

**Table 8:** Tensile mechanical test record of black fungus leaves.

Specimen	Tensile strength (N)	Tensile resistance (MPa)	Elastic modulus (MPa)	Poisson’s ratio	Shear modulus (MPa)
1	3.23	0.450	1.062	0.452	0.366
2	1.43	0.405	0.769	0.416	0.271
3	1.64	0.457	0.851	0.437	0.296
4	2.73	0.471	1.225	0.475	0.415
5	1.45	0.489	0.830	0.443	0.288
Mean	--	0.454	0.947	0.445	0.327
σ	--	0.028	0.070	0.019	0.055
cv	--	0.062	0.074	0.043	0.167

σ: Standard deviation. cv: Coefficient of variation.

We also found some similarities between the tensile strength at the base of the black fungus leaves and the rest. To prove this, we conducted a validation test. Select the mature black fungus 8 hours after water spray management, the measured moisture content is 84.2%, and the data are mixed in order from small to large, as shown in Table 9. The rank sum test was carried out, 14<T = 15<30. It was considered that there was no significant difference in tensile strength between the base of the black fungus leaves and the rest, and the tensile mechanical properties were consistent at the significant level α = 0.05.

**Table 9:** Rank sum test sample table.

	1	2	3	4	5	6	7	8	9
A	--	0.436	0.442	0.448	--	0.455	--	--	--
B	0.405	--	--	--	0.450	--	0.457	0.471	0.489

A: Base of the black fungus (MPa). B: Leaves of black fungus (MPa)

## Conclusions

In this paper, the black fungus of "Heishan" variety was selected as the research object, and the physical and mechanical properties of black fungus during the harvesting period were measured and analyzed. The main conclusions are as follows:

1. The height and thickness of the black fungus in the picking season conform to the normal distribution and are concentrated around 34.39mm and 0.92mm respectively; the average moisture content of the black fungus picked 8 hours after water spraying is 83.6%, and the average density is 1.091 g·cm^-3^.
2. A mono-factor separation test between black fungus and the sticks was designed, and the interference of the loading speed on the static separation force test was eliminated by the separation test of loading speed. Under quasi-static conditions, the separation force between black fungus and the fungus stick in the picking season was determined to be between 1.06N and 3.65N.
  When the tension angle was 0°, the average separation force was 3.167N. Through variance analysis, it can be concluded that there is no significant difference in the separation force between the black fungus and the sticks during the growth process, except for the significantly reduced separation force of over-mature black fungus. While the tension angle is an important influencing factor. In the measurement of the water content of black fungus during the picking season, It is recommended to harvest about 8 hours after water spray management, for drying process and other processing technique of black fungus.
3. In this experiment, we applied a new method to determine the Poisson’s ratio of black fungus by using high-speed photography and image processing technology to measure the displacement in the process of tensile test, with a test error below 2.5%. Through the comparison of the mechanical property data at the base of the black fungus leaves and the rest of leaves, it is concluded that the tensile properties of both are consistent. The test results show that the average tensile modulus of fresh black fungus is 0.947 MPa, the average value of the Poisson’s ratio is 0.445, and the average value of shear elastic modulus is 0.327 MPa.

Under the background of lack of researches on black fungus, the research methods and conclusions in this paper are of great significance for understanding the physical and mechanical properties of black fungus. The physical and mechanical properties of it provide theoretical support for the research and development of mechanized harvesting and processing equipment of black fungus, which will effectively improve the engineering level of mechanized harvesting and processing of black fungus, and promote the development of black fungus industry in the world.
